# Neural and behavioral responses to reproductive signals in male chorus frogs

**DOI:** 10.1242/jeb.251686

**Published:** 2026-04-20

**Authors:** Carlie B. Ochoa, Ashley M. Loeven, Debra Ann Fadool, Emily Moriarty Lemmon

**Affiliations:** ^1^ Department of Biological Science, Florida State University, 319 Stadium Drive, Tallahassee, FL 32306, USA; ^2^ Program in Neuroscience, Florida State University, Tallahassee, FL 32306, USA; ^3^ Institute of Molecular Biophysics, Florida State University, Tallahassee, FL 32306, USA

**Keywords:** Species recognition, Social decision-making network, pS6, Auditory midbrain, Acoustic signaling

## Abstract

Species recognition and courtship behaviors are powerful drivers of speciation. Here, we investigated the neural and behavioral signatures of species recognition in Upland chorus frogs (*Pseudacris feriarum*). Populations of this species that are sympatric with congeners (e.g. *Pseudacris nigrita*) have evolved divergent male mating calls and enhanced acoustic discrimination by females owing to costly interspecific hybridization. Herein, we examined evoked neural activity and behaviors in male *P. feriarum* in response to sympatric, allopatric or heterospecific calls, or silence, via phospho-S6 ribosomal protein immunofluorescence. The sympatric call evoked activity in several brain regions that regulate spatial navigation and social decision making, indicating that this call type may be an important trigger for navigating to and within a complex chorus environment. Moreover, each stimulus resulted in a unique pattern of coactivation among brain regions. Despite these neural changes, there were no differences in behavioral response to each stimulus. Our results suggest that signal input and behavioral output are coded independently in the brains of male chorus frogs. Together, these findings represent a first step towards understanding the neural basis of conspecific recognition in a system in which this trait contributes to ongoing diversification.

## Teaser

**Summary:** Neural and behavioral responses to acoustic stimuli in male *Pseudacris feriarum* indicate that signal input and behavioral output are coded independently in the brain, advancing our understanding of how animals recognize conspecifics.

## INTRODUCTION

The ability of an individual to recognize conspecifics mediates a diversity of social behaviors, including aggressive interactions and mate choices (Grether et al., 2009; Pfennig, 1998). In an evolutionary framework, this phenomenon of species recognition may, in some cases, define the boundary between species (Ryan and Rand, 1993). When interspecific interactions reduce the fitness of one or both actors, selection can enhance species recognition, strengthening the barrier between species (Howard, 1993). Despite their importance in evolutionary processes, the proximate mechanisms of species recognition – such as the biophysical and neural processes that regulate the detection and perception of conspecifics – remain relatively understudied beyond a small group of model organisms (e.g. Billeter et al., 2009; Araki et al., 2016; Mohr et al., 2018). Understanding the proximate mechanisms of species recognition can offer a window into how species are generated and maintained.

Certain properties of sexual signals can differ among closely related species, often serving as species identification cues (West-Eberhard, 1983; Ryan, 1990). Females use sexual signals that males produce to choose conspecific mates (Ptacek, 2000), and males use the same signals to choose worthwhile competitors (Grether et al., 2009) or as cues to adopt alternative mating strategies (e.g. satellite behavior; Humfield, 2008). Recent work has begun to describe the neural mechanisms that generate sexual signals (e.g. Hall et al., 2013; Tripp et al., 2021) and govern their perception in organisms such as midshipman fish (Mohr et al., 2018), *Heliconius* butterflies (Rossi et al., 2020) and gray treefrogs (Gupta et al., 2021).

Anuran amphibians offer a unique opportunity to study the neural mechanisms of species recognition, because most species show behavioral selectivity for conspecific vocalizations (termed ‘calls’ hereafter; Gerhardt, 1994). Typically, males produce advertisement calls to attract females and defend calling sites or territories (Wells, 1977). Females of most species show a strong preference for the calls of conspecific males (e.g. Gerhardt, 1994; but see Chen and Pfennig, 2020). Males may also recognize and preferentially respond to conspecific calls, in instances in which they can indicate the location of a breeding site and/or aid in avoiding costly interspecific aggression (Arak, 1983; Bee, 2007; Baugh and Ryan, 2010). Alternatively, males may be more permissive than females in their behavioral responses towards heterospecifics when there is less selective pressure on males to recognize and respond solely towards conspecifics (Bernal et al., 2007; González-Santoro et al., 2023).

Little is known about the neural basis of conspecific recognition in male anurans specifically, but previous work has revealed several key brain areas that may mediate auditory species recognition more generally. For example, immediate early gene expression in several auditory and forebrain areas differs in response to heterospecific versus conspecific calls (Hoke, 2004), and in response to attractive versus unattractive conspecific calls (Chakraborty et al., 2010). Of relevance are brain regions belonging to the vertebrate social decision-making network (SDMN), which includes regions involved in aggression, parental care and reproduction (O'Connell and Hofmann, 2012). In addition to the functional specialization of SDMN regions for species recognition, functional connectivity (e.g. synchronous activity among spatially separated brain regions) may also contribute to conspecific recognition (e.g. Hoke et al., 2005). Thus, SDMN nodes provide strong candidates for the neural basis of auditory species recognition in anurans.

Here, we measured the evoked patterns of functional neural activity in male Upland chorus frogs (*Pseudacris feriarum*). This species represents an incipient species radiation as different populations of *P. feriarum* sympatric with one or more congener (e.g. *Pseudacris nigrita*, *Pseudacris brimleyi*) undergo behavioral and genomic divergence (Lemmon, 2009; Banker et al., 2020). Sympatric populations have evolved calls with faster pulse rates and/or a greater number of pulses than have allopatric populations and *P. nigrita* (Lemmon, 2009). Female preferences for male calls have also evolved in sympatric populations such that these females discriminate against heterospecific calls and calls of *P. feriarum* males from populations allopatric with respect to *P. nigrita* (Lemmon, 2009). This enhanced species recognition in sympatric *P. feriarum* females generates a level of intraspecific reproductive isolation, representing an early stage in speciation (Lemmon, 2009). Several studies have assessed female acoustic preferences and their role in facilitating speciation in *P. feriarum* (e.g. Lemmon, 2009; Dye et al., 2024). Male acoustic preferences, however, have never been tested in this species, and it remains unclear whether, and to what extent, males display enhanced species recognition like *P. feriarum* females.

*Pseudacris feriarum* breed explosively in ephemeral ponds in which males call to attract females in very large choruses (Ethier et al., 2021). Sympatric male *P. feriarum* call alongside heterospecifics in breeding aggregations and may compete with heterospecifics for access to *P. feriarum* females, as evidenced by a prevalence of up to 2% F1 hybrids in some sympatric populations (Anderson et al., 2023). In deep sympatry (such as the field site described in this study), *P. feriarum* are relatively less abundant that *P. nigrita* (Lemmon, 2009), and, at any given breeding pond, males are many times more abundant than females (Ethier et al., 2021). This highly male-biased sex ratio at a breeding site can facilitate high levels of intermale competition (Kvarnemo and Ahnesjo, 1996), which can drive the evolution of competitor recognition (Grether et al., 2009; Moran and Fuller, 2018). Field observations confirm that calling male *P. feriarum* readily engage in physical combat if approached closely by another calling male (C.B.O. and E.M.L., personal observations). Satellite (non-calling) males are sometimes observed in the field in close physical proximity to a calling male (C.B.O. and E.M.L., personal observations). As in other hylids, it is believed that the proximity of another male prompts aggressive behaviors such as antiphonal calling and, if the intruder male continues to approach, physical combat (Rose and Brenowitz, 1991; Reichert and Gerhardt, 2014). Therefore, male *P. feriarum* may be subjected to selection for competitor recognition in sympatric populations (Grether et al., 2009). Here, we aimed to assess the extent to which sympatric male *P. feriarum* display species recognition at both neural and behavioral scale.

We conducted two experiments to accomplish our aim. In the evoked neural activity trials, we exposed sympatric male *P. feriarum* to one of four acoustic stimuli (sympatric *P. feriarum* call, allopatric *P. feriarum* call, *P. nigrita* call and silence) and collected the brains to (1) determine which SDMN nodes show evoked neural activity patterns that could contribute to species recognition, and (2) test for a role of functional connectivity among SDMN nodes in species recognition. In the male behavior trials, we scored sympatric male *P. feriarum* behavior in response to each of the four acoustic stimuli in an arena allowing for free movement to (3) assess how evoked patterns of neural activity might translate into ecologically and evolutionarily relevant behaviors in male chorus frogs. The results of this study provide valuable insight into the neural mechanisms that govern how animals recognize conspecifics and represent one of the first studies to examine the neural basis of ongoing evolutionary diversification.

## MATERIALS AND METHODS

### Ethics

All procedures described here were approved by the Florida State University Animal Care and Use Committee (#PROTO202000029) and the American Veterinary Medical Association. All anesthetic and euthanasia procedures met published guidelines for the ethical treatment of animals. A research permit to collect frogs from publicly owned lands was issued by the Florida Fish and Wildlife Conservation Commission (permit number LSSC-22-00019).

### Animal collection

We captured 35 adult male *Pseudacris feriarum* Baird 1854 by hand in a natural breeding pond in Apalachicola National Forest (Liberty County, FL, USA) in January 2024 (these frogs were used in the evoked neural activity trials) and 24 additional adult male *P. feriarum* from the same pond in February 2025 (these frogs were used in the male behavior trials; Fig. S1). For an initial time course experiment, we sampled six adult male *P. feriarum* in February 2023 from the same pond using the same methods. All males were observed calling before capture. In this locality, the focal species coexists with the congener *P. nigrita* and has evolved distinct male calls and female preferences (Lemmon, 2009). We collected only males for all experiments described here to avoid depleting the site of gravid females and thus preserve the local population. We immediately transported all animals from the field site to the Florida State University campus in Tallahassee, FL. We kept all frogs chilled (4°C) and individually housed in a dark, quiet environment for 8–12 h following capture before evoked neural activity trials and for 8–48 h following capture before male behavior trials. We deemed this acclimation period sufficient to reduce signatures of neural activity induced by chorusing at the field site because a frog's immediate early gene mRNA declines to very low levels within 2–4 h in isolation after capture from a chorus (Burmeister et al., 2008).

### Acoustic stimuli

Each of the three acoustic stimuli were synthesized previously and shared with permission from the authors of Dye et al. (2024). The stimuli consisted of the focal call representing the average call for a given call type, repeated every second overlaid upon background chorus noise. Because the temporal characteristics of calls vary with temperature, all stimuli were corrected to represent the average pulse rate and pulse number of the call type at 20°C. The same stimuli were used in the experiments described in the evoked neural activity trials, time course experiment and male behavior trials.

### Evoked neural activity trials

**Fig. 1.**
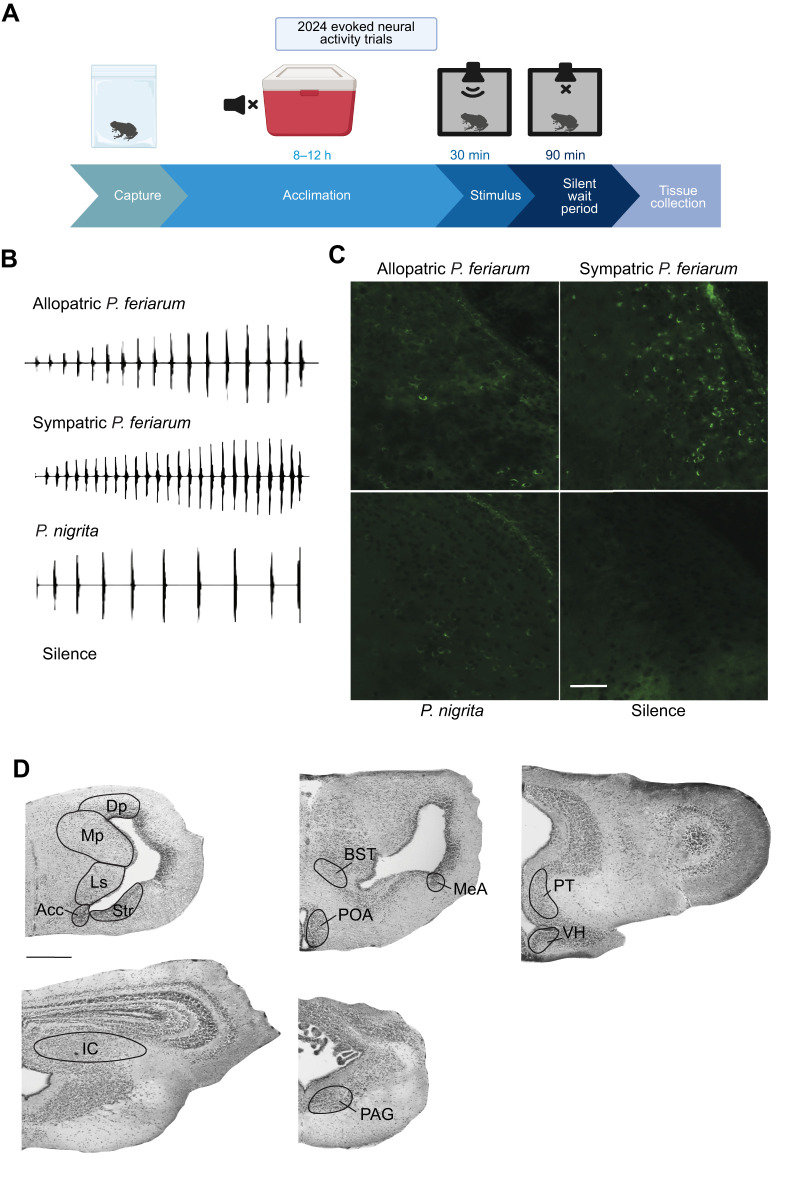
**Evoked neural activity trials in *Pseudacris feriarum*.** (A) Experimental procedure and timeline for evoked neural activity trials. (B) Oscillograms of the four acoustic stimuli used in the evoked neural activity trials and male behavior trials. (C) Representative photomicrographs of the lateral septum that display a predicted pattern of phospho-S6 ribosomal protein (pS6) induction for each of the four stimuli. Scale bar: 50 µm. Overall brightness was increased to improve visibility in consultation with the journal. (D) Brightfield images of Nissl-stained coronal sections indicating the locations of each of the 12 brain regions sampled. Acc, nucleus accumbens; BST, bed nucleus of the stria terminalis; Dp, dorsal pallium; IC, inferior colliculus; Ls, lateral septum; MeA, medial amygdala; Mp, medial pallium; PAG, periaqueductal gray; POA, anterior preoptic area; PT, posterior tuberculum; Str, striatum; VH, ventral hypothalamus. Scale bar: 500 µm.

We conducted acoustic stimulation trials to examine the differences in evoked neural activity among *P. feriarum* males exposed to different acoustic stimuli (Fig. 1). For these trials, we randomly assigned males to one of four treatment groups: the sympatric advertisement call, the allopatric advertisement call, the *P. nigrita* advertisement call (*n*=9 for each group) or silence (*n*=8). We chose to conduct this experiment separately from the behavioral experiment to assess neural activity induced by sound exposure, rather than by movement in a larger testing arena (Hoke et al., 2007).

For each trial, we transferred a male directly from the 4°C chamber into a small plastic dish covered in mesh, with a knot of vegetation. We placed the frog in the tub into a custom, temperature-controlled, acoustically insulated chamber (hereafter, ‘box’). Inside each box, the plastic dish rested in a small plastic tub filled with dechlorinated water to simulate the natural environment of a breeding pond. Inside the container, the frog could rest with its body two-thirds submerged in water, as it would while calling in a breeding pond. We used a thermometer to monitor water temperature inside the box before and during all trials and manually adjusted the temperature as necessary to maintain a water temperature of 20±1°C at all times during the trials. *Pseudacris feriarum* body temperature adjusts to water temperature with a 30–60 s delay; thus, the males were able to achieve a body temperature of 20°C soon after being placed in the box.

In each box, above the frog, we placed a small, Bluetooth speaker (Ewa Audio A106 Pro) to play the acoustic stimulus. Before the trials, all speakers were equilibrated to a sound intensity level of 75 dB, which approximates the intensity of a chorus frog advertisement call at ∼1 m, simulating the natural chorus environment with a calling neighbor. For all treatment groups, we played the assigned stimulus for 30 min, then left the male in silence in the box for an additional 90 min, resulting in a total of 120 min elapsing between the stimulus start time and the time of tissue collection (Fig. 1). We used this timing regime because of the results of the time course experiment described below. During the stimulation period, we recorded whether each male called in response to the stimulus.

After the post-stimulation period had elapsed, we quickly removed the animal from the box and anesthetized it with application of 20% benzocaine gel (Orajel brand) to the belly. After waiting 1–3 min to allow the anesthetic to take effect, we rapidly decapitated the animal, removed extraneous tissue from the head and transferred the entire brain into chilled 4% paraformaldehyde (PFA) in 1× phosphate buffered saline (PBS) containing 137 mmol l^−1^ NaCl, 2.7 mmol l^−1^ KCl, 10 mmol l^−1^ Na_2_HPO_4_ and 1.8 mmol l^−1^ KH_2_PO_4_ (Tucker and Fadool, 2002). From the time of removal from the box to completion of the dissection, no more than 5 min had elapsed. All sacrifices took place during daytime hours (between 10:00 h and 15:30 h) in a darkened room the day after capture from the field to minimize circadian biases.

### Immunofluorescent labeling

All brains remained in 4% PFA/PBS at 4°C for 12–20 h before three 5-min PBS washes and cryoprotection. Brains were cryoprotected with 10% sucrose in PBS for 24 h at 4°C, then with 30% sucrose in PBS for 24–48 h at 4°C; both incubations took place in a static environment. Following cryoprotection, brains were snap frozen and embedded in Tissue Tek O.C.T. Compound (Sakura Finetek). Brains were stored at −80°C until they were sectioned.

Brains were coronally sectioned (Microm HM525 cryostat, Thermo Fisher Scientific) at 20 µm thickness in two series and transferred to 2% gelatine-coated slides. Prepared cryosections were stored at −20°C until processing for immunolabeling. One series was used for immunofluorescent labeling to avoid double counting cells. Here, slides were washed in PBS and then blocked in a solution of 5% normal goat serum in PBS-T (0.03% Triton X-100 in PBS) for 1 h at room temperature. Slides were incubated in 1:500 anti-phospho-S6 ribosomal protein (pS6) antibody (2211, Lot #24, Cell Signaling Technology) in 2% normal goat serum in PBS-T overnight at 4°C. The next day, slides were washed in PBS and then incubated in 1:200 species-specific secondary antibody (AlexaFluor488, A11034, Lot #2752650, Invitrogen) in 2% normal goat serum in PBS-T for 2 h at room temperature in the dark. Secondary antisera were washed with PBS, then slides were coverslipped using Vectashield with DAPI (H-1200-10, Vector Laboratories) and stored at −20°C until imaging. The anti-pS6 antibody detects endogenous levels of ribosomal protein S6 when phosphorylated at Ser235 and Ser236. This polyclonal antiserum was produced by immunizing rabbits with a synthetic phosphopeptide surrounding residues Ser235 and Ser236 using human ribosomal protein S6 and was then affinity purified (Flotow and Thomas, 1992). The specific antibody we used has been successfully shown to approximate neural activity in a variety of species, including other anurans (e.g. Fischer et al., 2018, 2019, 2020).

### Microscopy and cell counting

Brain sections were imaged on a Keyence BZ-X710 High Resolution Fluorescence Microscope at 20× magnification with a 488 nm wavelength light source. In total, pS6-positive cells were quantified across 12 brain regions: the nucleus accumbens (Acc), dorsal pallium (Dp), inferior colliculus (IC), striatum (Str), medial pallium (Mp), anterior preoptic area (POA), lateral septum (Ls), periaqueductal gray (PAG), posterior tuberculum (PT), ventral hypothalamus (VH), bed nucleus of the stria terminalis (BST) and medial amygdala (MeA; Fig. 1). We counted all pS6-positive cells in each region for one hemisphere (randomly selected) for every section in which the region was clearly identifiable. DAPI nuclear staining and a draft chorus frog brain atlas were used to identify brain regions and quantify the number of pS6-positive cells from images using ImageJ software (Fig. 1; Schneider et al., 2012). Note that the photomicrographs shown in Fig. 1C were edited to increase the overall brightness of the images for ease of viewing with permission from the journal and in accordance with their image manipulation policy.

### Regional analysis

**Fig. 2.**
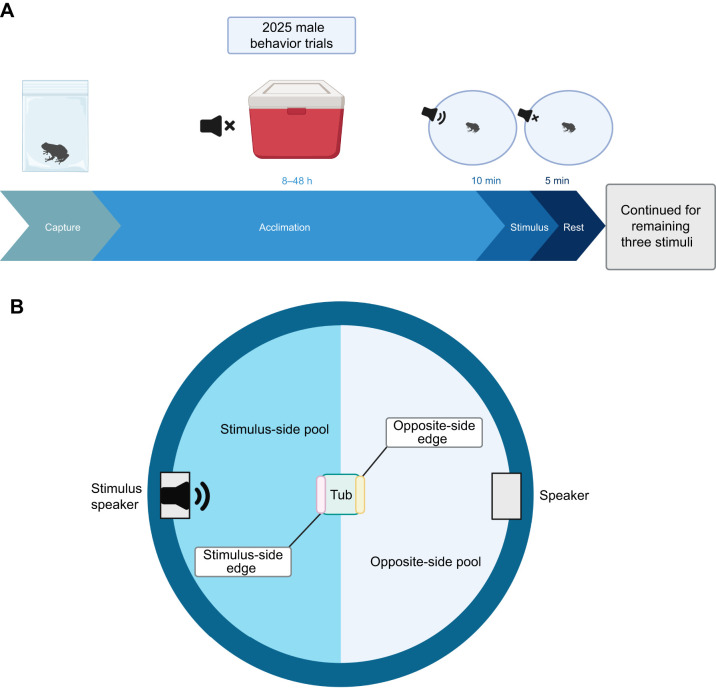
**Male behavior trials in *P. feriarum*.** (A) Experimental procedure and timeline for male behavior trials. (B) The male behavior arena (top-down view), with areas scored for positional behaviors marked. For each male behavior trial, the frog started in the central tub (green) and was allowed to move freely in the arena for 10 min while a stimulus played from one speaker. We scored the duration of perching on an edge of the tub (either the stimulus-facing side, marked in pink, or the opposite side, marked in yellow) and time spent on either side of the pool (stimulus facing, marked in blue, or the opposite side, marked in light gray-blue).

We analyzed the relationship between acoustic stimulus type and pS6 neural activity in each focal brain region separately to identify the regions that showed acoustically evoked patterns of neural activity indicative of enhanced species recognition. We conducted all statistical analyses in R version 4.4.2 (https://www.r-project.org/). For each region, we constructed a generalized linear mixed model (GLMM) with a negative binomial distribution to test for differences in pS6-positive cell number between stimulus groups. The negative binomial distribution is appropriate for this dataset because it consists of count data with unequal variances. For each region, we used a GLMM with stimulus group and antiphonal calling (presence or absence) as main effects, brain region area as an offset variable and, where possible, animal identity as a random effect to account for the repeated measures design of the experiment (multiple sections imaged per region per individual). For some brain regions, the random effects of animal identity approached zero (on the order of 10^−10^), which caused the model to fail to converge. For these regions, we instead used a generalized linear model (GLM) with a negative binomial distribution with the same main effects and offset variable. These regions were the Acc, IC, Str, POA, BST, MeA and PT. We performed model comparison for each region to determine whether an additive or interactive model better fit the data. We further explored the main effects of group and antiphonal calling on pS6-positive cell counts with *post hoc* analyses using pairwise comparisons of estimated marginal means with *P*-values adjusted using Tukey's honestly significant difference corrections (emmeans package; https://rvlenth.github.io/emmeans/). We predicted that, for regions with functional specialization for enhanced species recognition, (1) the number of pS6-positive cells would be increased (or decreased) in response to the sympatric stimulus, compared with all other stimuli, (2) the number of pS6-positive cells would not differ between the heterospecific and the allopatric stimulus treatments, and (3) the number of pS6-positive cells would differ between the silent treatment group and each acoustic stimulus group (Fig. 2).

### Coactivation analysis

We tested for differences in brain region coactivation patterns between stimulus groups to identify the connections among SDMN regions that may contribute to enhanced species recognition. If coactivation patterns among SDMN regions contributed to enhanced species recognition, we predicted that the patterns induced by the sympatric *P. feriarum* stimulus should be unique compared with those of the other stimuli. To analyze coactivation patterns, we computed Pearson correlation coefficients for the number of pS6-positive cells between each pair of SDMN regions within each stimulus group. To compare correlation matrices between stimulus groups, we used the quadratic assignment procedure (QAP) test with 5000 permutations in the R package sna (https://CRAN.R-project.org/package=sna) following Teles et al. (2015). The null hypothesis for this test is that the two input matrices are different from one another. Therefore, *P*≤0.05 indicates that the matrices are not different from one another. To further explore differences in coactivation patterns between stimulus groups, we conducted correlation tests for the number of pS6-positive cells for each pairwise combination of brain regions and qualitatively compared the significant (*P*≤0.05) pairwise associations among stimulus groups.

### Time course experiment

To determine the time course of pS6 induction in the *P. feriarum* brain, we conducted an initial experiment comparing the number of pS6-positive cells 60 min and 120 min after acoustic stimulation. For these trials, we randomly assigned males to one of three treatments: silence, sympatric call with 60 min between the onset of the stimulus and tissue collection, or sympatric call with 120 min between the onset of the stimulus and tissue collection (*n*=2 for each group). We used the same acoustic stimulation procedure as described in the evoked neural activity trials, except that, following the 30-min stimulus period, we left each male to remain in the box for either an additional 30 or 90 min according to the male's assigned treatment group. For frogs assigned to the silence group, we left the frogs in the box for 60 min in total. After each frog's time in the box had elapsed, we quickly removed the frog from the box and proceeded with the same tissue collection, pS6 immunofluorescence, microscopy and cell counting procedure described for the evoked neural activity trials. We tested for a difference in the number of pS6-positive cells between the time groups (data from all brain regions pooled together) using Wilcoxon rank sum tests.

### Male behavior trials

We conducted an experiment to assess how any neural differences evoked by the four stimulus types might translate to behavior. For these trials, each frog (*n*=24) was exposed to each of the following acoustic stimuli used in the evoked neural activity trials in a random order and with 5-min breaks between trials (Fig. 2). We constructed a testing arena by placing a 1.2-m diameter plastic pool filled with ∼7.5 cm of 20°C dechlorinated water inside a soundproof chamber. Throughout trials, we monitored water temperature constantly and manually maintained the temperature at 20°C±1°C by adding ice or hot water. We placed a bamboo grid at the water's surface to facilitate the frog's movement and positioned speakers (Mineroff SME-AFS Amplified Field Speaker) on opposing sides. We equilibrated the sound pressure level of each speaker to 75 dB sound pressure level, as in the evoked neural activity trials. We also placed a small plastic tub filled with leaf litter from the field site and dechlorinated water in the center of the pool to serve as a starting point for each trial. For each trial, we placed a frog inside this tub and let it remain in the tub in silence for 5 min. We then began remote playback of one of the four acoustic stimuli from one speaker with background chorus noise from the opposite speaker, and we recorded a video of the frog's behavior for 10 min (Sony Handycam HDR-SR11). We chose this length of time because similar studies have recorded male anuran behavior in response to acoustic stimuli for periods ranging from 5 to 15 min (e.g. Bee, 2007; Baugh and Ryan, 2010; González-Santoro et al., 2023), and initial exploratory trials indicated that male *P. feriarum* behavior did not change after 8–10 min. After the trial period, we stopped the playback, placed the frog back in the tub and let the frog acclimate for an additional 5 min in silence before beginning a new trial using another stimulus. We repeated this procedure until each frog had completed a trial for each of the four stimuli (Fig. 2). For each trial, we randomized which speaker played the stimulus and the order of trials to control for directional biases and precedence effects, respectively. After each frog had completed all four trials, we measured the frog's body mass and snout vent length (SVL) using digital calipers.

### Behavioral analysis

We scored the videos from the male behavior trials using BORIS version 9.3.5 (Friard and Gamba, 2016). We recorded the total duration of two behaviors – calling and movement – as well as positional behaviors relative to the speaker with stimulus playback. Scorers were unaware of the identity of call types. For positional behaviors, we scored the duration of perching on an edge of the tub (either the stimulus-facing side or the opposite side) and time spent on either side of the pool (stimulus-facing or opposite side; Fig. 2). We chose to score spatial position in this way because we aimed to assess broad patterns of affiliation rather than ‘choice’ as in a traditional phonotaxis assay. We tested for differences in the duration of behaviors among the four stimulus treatments by constructing a linear mixed effect model (R packages lme4 and lmerTest; Bates et al., 2015; Kuznetsova et al., 2017). We coded the stimulus type and behavior type as interactive fixed effects with a random effect of frog identity. Then, we conducted an ANOVA using the Kenward–Roger degrees of freedom calculation for analysis of model fixed effects and pairwise *post hoc* tests to analyze interactive effects (emmeans package). All *P*-values were Tukey adjusted for multiple hypothesis testing. For positional behaviors as we define them (relative to the stimulus speaker), we omitted the silence treatment in *post hoc* tests because there was no speaker with stimulus playback during these tests.

We similarly tested for an effect of body size and trial number (independent of stimulus) on all behavioral outcomes by constructing linear mixed effect models with the same effect structure but including an additional interactive term of either SVL, body mass or trial number. We then assessed which model fit better with corrected Akaike's information criterion (AICc) model comparison (R package AICcmodavg; https://cran.r-project.org/package=AICcmodavg) to determine whether SVL, body mass or trial number affects the duration of the behaviors we recorded.

## RESULTS

### Time course of pS6 induction

We found greater neural activity in the 120-min treatment group than in the 60-min treatment group and a silence control group (Fig. S2). Frogs that were allowed 120 min after the stimulus start time had more pS6-positive cells than frogs that were allowed 60 min (Wilcoxon rank sum test; *W*=2273.5, *P*=0.03) or frogs that did not receive any acoustic stimulation (Wilcoxon rank sum test; *W*=2065, *P*=0.0009; Fig. S2).

### Evoked neural activity in SDMN regions

**Fig. 3.**
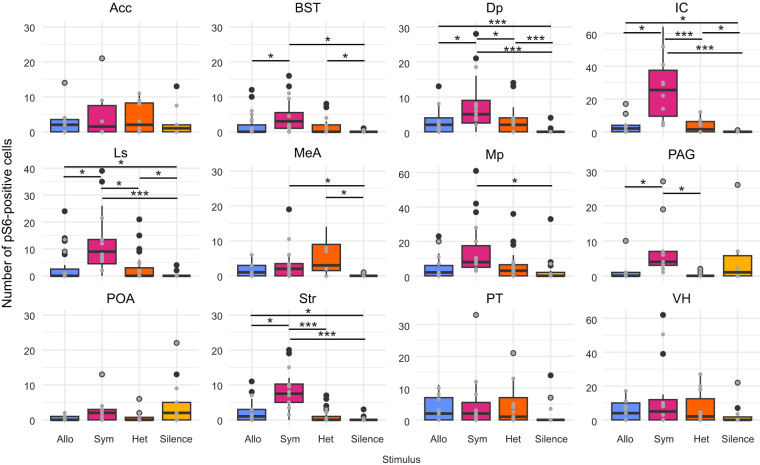
**pS6-positive cell counts for each of 12 brain regions sampled in *P. feriarum* across the four stimulus groups.** *n*=9 allopatric *P. feriarum* (blue); *n*=9 sympatric *P. feriarum* (magenta); *n*=9 heterospecific *P. nigrita* (orange); *n*=8 silence (yellow). Allo, allopatric *P. feriarum* stimulus; Sym, sympatric *P. feriarum* stimulus; Het, *P. nigrita* stimulus. In each box plot, interquartile range and median are shown by boxes with a horizontal line, respectively. Black points represent data points lying more than 1.5× the interquartile range from the median. Gray points represent median cell counts per individual. Whiskers indicate 1.5× the interquartile range from the box's upper boundary (Q3) or lower boundary (Q1). Horizontal lines with asterisks indicate significant (*P≤0.05) and highly significant (***P<0.0001) pairwise comparisons (Table 1).

Four of the brain regions we sampled met the predicted criteria for functional specialization relating to enhanced species recognition (sympatric stimulus results in different number of pS6-positive cells than all other treatments; *P. nigrita* and allopatric stimuli result in the same number of pS6-positive cells). These regions were the Dp, IC, Ls and Str (Table 1, Fig. 3). There was no effect of antiphonal calling or time of dissection on the number of pS6-positive cells for any region sampled. For all regions except the IC, the additive model provided a better fit than the interactive model. However, for the IC model, none of the interactions between stimulus type and antiphonal calling were significant. Several regions did not have any significant main effects of either stimulus type or calling in response on pS6-positive cell counts (Table 1).

**Table 1.** Pairwise *post hoc* contrasts of stimulus types for each brain region sampled in *Pseudacris feriarum*

Region	Stimulus group pair	Estimate (s.e.)	*z*-ratio	*P*
Acc	Allo–Sym	−0.161 (0.91)	−0.177	0.998
	Allo–Het	−0.425 (0.84)	−0.502	0.958
	Allo–Silence	0.205 (0.87)	0.233	0.995
	Sym–Het	−0.264 (0.87)	−0.300	0.990
	Sym–Silence	0.366 (0.39)	0.397	0.978
	Het–Silence	0.630 (0.84)	0.749	0.877
POA	Allo–Sym	−1.568 (0.88)	−1.768	0.288
	Allo–Het	−0.653 (0.90)	−0.720	0.889
	Allo–Silence	−1.972 (0.87)	−2.258	0.648
	Sym–Het	0.915 (0.78)	1.165	0.952
	Sym–Silence	−0.404 (0.76)	−0.529	0.952
	Het–Silence	−1.319 (0.77)	−1.700	0.323
BST	Allo–Sym	−0.583 (0.55)	−1.055	0.716
	Allo–Het	0.264 (0.55)	0.534	0.950
	Allo–Silence	3.326 (1.1)	3.020	0.013*
	Sym–Het	0.877 (0.56)	1.547	0.409
	Sym–Silence	3.910 (1.1)	3.517	0.002*
	Het–Silence	3.032 (1.1)	2.746	0.030*
Dp	Allo–Sym	−1.034 (0.34)	−3.040	0.012*
	Allo–Het	−0.123 (0.33)	−0.365	0.983
	Allo–Silence	2.504 (0.52)	4.751	<0.0001*
	Sym–Het	0.912 (0.34)	2.652	0.039*
	Sym–Silence	3.539 (0.53)	6.657	<0.0001*
	Het–Silence	2.627 (0.52)	4.979	<0.0001*
PAG	Allo–Sym	−2.25 (0.76)	−2.933	0.017*
	Allo–Het	1.51 (0.93)	1.616	0.369
	Allo–Silence	−0.63 (0.83)	−0.751	0.876
	Sym–Het	3.77 (0.91)	4.112	0.0002*
	Sym–Silence	1.62 (0.81)	1.987	0.193
	Het–Silence	−2.14 (0.97)	−2.201	0.123
IC	Allo–Sym	−2.360 (0.56)	−4.146	0.0002*
	Allo–Het	0.251 (0.59)	0.420	0.975
	Allo–Silence	2.725 (0.94)	2.888	0.020*
	Sym–Het	2.611 (0.56)	4.631	<0.0001*
	Sym–Silence	5.085 (0.92)	5.479	<0.0001*
	Het–Silence	2.474 (0.93)	2.637	0.041*
Ls	Allo–Sym	−1.643 (0.53)	−3.053	0.012*
	Allo–Het	−0.013 (0.50)	−0.027	1.000
	Allo–Silence	2.178 (0.68)	3.201	0.007*
	Sym–Het	1.630 (0.52)	3.119	0.009*
	Sym–Silence	3.822 (0.72)	5.309	<0.0001*
	Het–Silence	2.192 (0.68)	3.222	0.007*
MeA	Allo–Sym	−0.392 (0.57)	−0.681	0.904
	Allo–Het	−1.031 (0.60)	−1.717	0.314
	Allo–Silence	2.594 (1.1)	2.229	0.115
	Sym–Het	−0.639 (0.49)	−1.285	0.572
	Sym–Silence	2.987 (1.1)	2.675	0.037*
	Het–Silence	3.625 (1.1)	3.219	0.007*
Mp	Allo–Sym	−0.953 (0.49)	−1.921	0.219
	Allo–Het	0.184 (0.49)	0.376	0.981
	Allo–Silence	1.277 (0.54)	2.363	0.084
	Sym–Het	1.137 (0.50)	2.254	0.109
	Sym–Silence	2.230 (0.55)	4.019	0.0003*
	Het–Silence	1.093 (0.53)	2.036	0.174
Str	Allo–Sym	−1.623 (0.38)	−4.249	0.0001*
	Allo–Het	0.641 (0.40)	1.603	0.376
	Allo–Silence	1.901 (0.54)	3.514	0.0025*
	Sym–Het	2.265 (0.41)	5.530	<0.0001*
	Sym–Silence	3.525 (0.55)	6.363	<0.0001*
	Het–Silence	1.260 (0.56)	2.240	0.112
PT	Allo–Sym	−0.428 (0.73)	−0.582	0.937
	Allo–Het	−0.522 (0.73)	−0.715	0.891
	Allo–Silence	0.412 (0.80)	0.513	0.956
	Sym–Het	−0.094 (0.72)	−0.129	0.999
	Sym–Silence	0.841 (0.80)	1.042	0.724
	Het–Silence	0.935 (0.79)	1.171	0.645
VH	Allo–Sym	−0.261 (1.1)	−0.223	0.996
	Allo–Het	−0.470 (1.1)	−0.416	0.975
	Allo–Silence	1.209 (1.3)	0.906	0.801
	Sym–Het	−0.210 (1.1)	−0.183	0.997
	Sym–Silence	1.470 (1.3)	1.076	0.704
	Het–Silence	1.680 (1.3)	1.256	0.591

‘Estimate’ represents the difference between estimated marginal means for the stimulus pair. Significant Tukey-adjusted *P*-values are marked with asterisks (*P≤0.05). Acc, nucleus accumbens; Allo, allopatric *P. feriarum* stimulus; BST, bed nucleus of the stria terminalis; Dp, dorsal pallium; Het, *P. nigrita* stimulus; IC, inferior colliculus; Ls, lateral septum; MeA, medial amygdala; Mp, medial pallium; PAG, periaqueductal gray; POA, anterior preoptic area; PT, posterior tuberculum; Str, striatum; Sym, sympatric *P. feriarum* stimulus; VH, ventral hypothalamus.

### Coactivation of SDMN regions

**Fig. 4.**
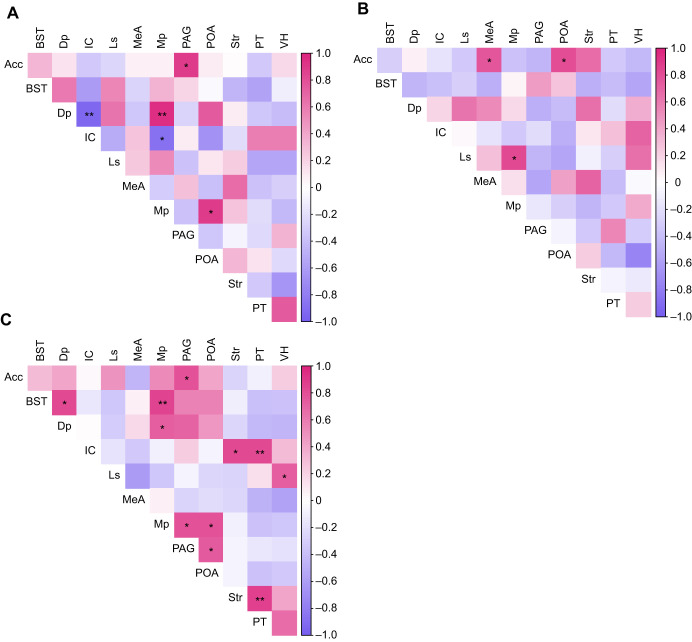
**Correlation heatmaps of brain region coactivation in *P. feriarum*.** (A–C) Regional correlations for frogs exposed to the sympatric *P. feriarum* stimulus (*n*=9; A), allopatric *P. feriarum* stimulus (*n*=9; B) and *P. nigrita* stimulus (*n*=9; C). Cells on each heatmap are colored by the strength of the correlation (see scale bar on the right of each plot) with significant correlations marked with asterisks (*P≤0.05, **P<0.01). *P*-values for each pair of regions for each stimulus group are shown in Table S1.

pS6 immunoreactivity revealed unique coactivation patterns in response to all three acoustic stimuli. QAP tests demonstrated a very weak positive relationship between each combination of matrices: the allopatric and sympatric matrices (*r*=0.057, *P*>0.05), the sympatric and heterospecific matrices (*r*=0.186, *P*>0.05), and the allopatric and heterospecific matrices (*r*=0.028, *P*>0.05; Table 2). Because all *P*-values were greater than 0.05, each matrix was different from one another, indicating that each stimulus results in a unique coactivation pattern. We detected many significant pairwise correlations in the number of pS6-positive cells between brain regions in all acoustic stimuli groups (Fig. 4). The sympatric stimulus resulted in five significant pairwise correlations, two of which were unique to this group: Dp–IC and Mp–IC. The allopatric stimulus resulted in three significant pairwise correlations, all of which were unique to this group: Acc–MeA, Acc–POA and Ls–Mp. Lastly, the heterospecific stimulus resulted in 11 significant pairwise correlations, seven of which were unique: BST–Dp, IC–Str, IC–PT, Ls–VH, Mp–PAG, PAG–POA and Str–PT (Fig. 4, Table S1). Only three significant pairwise correlations were shared between more than one stimulus group: Acc–PAG, Dp–Mp and Mp–POA correlations were significant in both the sympatric and heterospecific stimulus groups. We excluded data from frogs in the silence stimulus group from the QAP and correlation tests because the pS6-positive cell count data from this group were zero inflated, preventing transformation of the data into matrix format.

**Table 2.** Quadratic assignment procedure test output for comparison of coactivation patterns among brain regions in *P. feriarum* for each pair of sound stimuli*

Matrix comparison	Correlation	*P* (positive effect)	*P* (negative effect)
Sym–Allo	0.057	0.270	0.729
Sym–Het	0.186	0.063	0.936
Allo–Het	0.028	0.355	0.645

*The correlation between the two input matrices is expressed as the observed test statistic for the quadratic assignment procedure (QAP) test. *P* (positive effect) is the proportion of permuted test statistics that were greater than or equal to the observed data. Likewise, *P* (negative effect) is the proportion of permuted test statistics that were less than or equal to the observed data. Because the null hypothesis for each test is that the input matrices are different from one another, *P*-values greater than 0.05 confirm the dissimilarity of the matrices.

### Male behavior

**Fig. 5.**
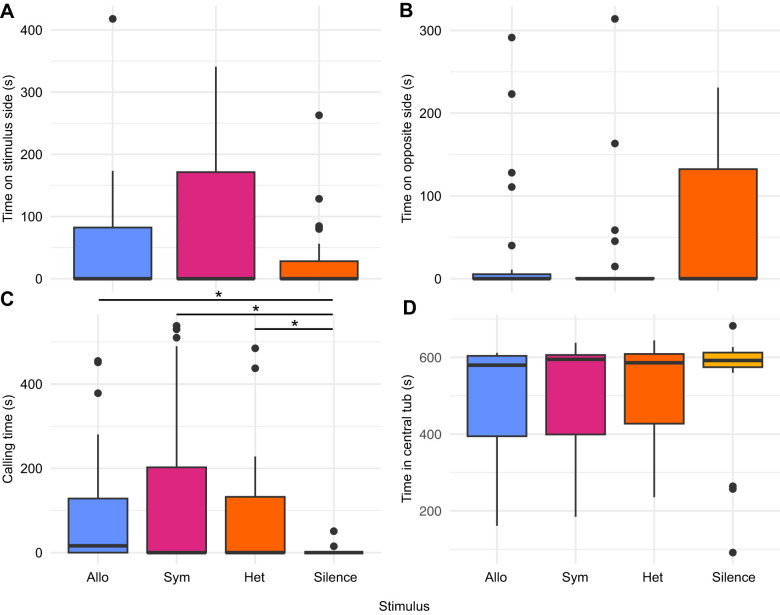
**Results of four key behavior metrics scored in male behavior trials in *P. feriarum*.** Allopatric *P. feriarum* (blue); sympatric *P. feriarum* (magenta); heterospecific *P. nigrita* (orange); silence (yellow). (A) Total time spent in the stimulus-facing side of arena (including edge of tub and in pool). (B) Total time spent in the opposite side of the arena (including edge of tub and in pool). (C) Total time spent advertisement calling. Horizontal lines with asterisks indicate significant (*P≤0.05) pairwise comparisons (Table 3). (D) Total time spent inside the central starting tub. *n*=22 frogs were tested once in each stimulus condition. In each box plot, interquartile range and median are shown by boxes with a horizontal line, respectively. Black points represent data points lying more than 1.5× the interquartile range from the median. Whiskers indicate 1.5× the interquartile range from the box's upper boundary (Q3) or lower boundary (Q1).

Frog behavior largely did not differ across the four stimuli trials (Fig. 5). The only exception was that frogs called more in response to any of the sound stimuli than to silence, but the total duration of calling did not differ among the sympatric, allopatric and heterospecific groups (Table 3). There were no differences among the stimuli groups in any positional behavior (e.g. total duration in central tub, duration on the side of the arena with stimulus playback, etc.) or in time spent in motion (Tables 3 and 4, Table S2). Additionally, there was no main effect of SVL, body mass or trial number on the duration of any behavior for any stimulus group (Table S3). We excluded two frogs from the final analysis owing to abnormal behavior (immediate escape from arena in all trials; final *n*=22).

**Table 3.** Pairwise *post hoc* contrasts of stimulus groups for each scored behavior in *P. feriarum*

Contrast	*t*	*P*
Calling		
Allo–Sym	−1.305	0.559
Allo–Het	0.518	0.954
Allo–Silence	3.747	0.001*
Sym–Het	1.823	0.263
Sym–Silence	5.052	<0.001*
Het–Silence	3.229	0.007*
Duration spent in central tub		
Allo–Sym	0.223	0.996
Allo–Het	−0.744	0.879
Allo–Silence	−1.725	0.311
Sym–Het	−0.967	0.768
Sym–Silence	−1.948	0.209
Het–Silence	−0.981	0.760
Movement duration		
Allo–Sym	0.051	1.000
Allo–Het	−0.070	0.999
Allo–Silence	0.389	0.980
Sym–Het	−0.120	0.999
Sym–Silence	0.338	0.986
Het–Silence	0.458	0.968
Duration spent on edge of central tub facing stimulus		
Allo–Sym	−0.748	0.735
Allo–Het	−0.550	0.846
Sym–Het	0.198	0.978
Duration spent in pool on side of arena with stimulus		
Allo–Sym	−0.436	0.900
Allo–Het	1.247	0.426
Sym–Het	1.682	0.212
Total duration spent on side of arena with stimulus		
Allo–Sym	−2.00	0.112
Allo–Het	0.293	0.953
Sym–Het	2.320	0.053
Duration spent on edge of central tub facing opposite the stimulus		
Allo–Sym	−0.325	0.943
Allo–Het	−0.597	0.821
Sym–Het	−0.272	0.960
Duration spent in pool on side of arena opposite the stimulus		
Allo–Sym	0.666	0.783
Allo–Het	−0.085	0.996
Sym–Het	−0.751	0.733
Total duration spent on side of arena opposite the stimulus		
Allo–Sym	0.341	0.937
Allo–Het	−0.734	0.743
Sym–Het	−1.075	0.529

*Significant Tukey-adjusted *P*-values are marked with asterisks (*P≤0.05). d.f.=637 for all contrasts. We were unable to include contrasts with the Silence stimulus group for positional behaviors that we scored relative to the stimulus speaker, because this treatment did not have a stimulus-playing speaker.

**Table 4.** Summary of the main effects of the generalized linear model comparing effects of stimulus type and behavior type on behavior duration in *P. feriarum*

Effects	*F*	d.f.	*P*
Stimulus	1.879	3, 637	0.131
Behavior	285.9	8, 637	2.2×10^−16^
Stimulus×behavior	2.174	18, 637	0.003

## DISCUSSION

We aimed to assess behavioral and neural signatures of enhanced species recognition in male *P. feriarum* by examining functional evoked neural activity and observing behavior. We found that three SDMN nodes as well as the auditory midbrain show evoked neural activity patterns consistent with our prediction of specialization for enhanced species recognition (Fig. 3). We also found that each of the three sound-based stimuli (sympatric *P. feriarum* call, allopatric *P. feriarum* call, *P. nigrita* call) produced unique patterns of coactivation among SDMN nodes (Fig. 4), indicating a role for functional connectivity in generating unique neural responses to each stimulus. These results indicate a role for functional connectivity in perception of the sympatric *P. feriarum* call and the heterospecific call. The heterospecific call group showed many more significant and unique pairwise correlations than the other two call type groups. Only three significant pairwise correlations were shared between more than one stimulus group, and these were all shared between the sympatric and heterospecific groups. Despite differences in evoked neural activity patterns within and between brain regions, we did not find any significant differences in positional behaviors or time spent in motion among acoustic stimuli treatments (Fig. 5, Table 3). Our behavioral results do show, however, that, at a sound intensity level equal to that of a calling neighbor at ∼1 m, male *P. feriarum* do not readily demonstrate directed phonotaxis but, rather, spend most of the trial time calling in place.

### Specialization of brain regions for enhanced species recognition

Specialization of the IC, Dp, Ls and Str likely contributes to enhanced species recognition in *P. feriarum*. These four regions show greater pS6 induction in response to the sympatric *P. feriarum* call than to all other stimuli, and no difference in pS6-positive cell count in response to the allopatric *P. feriarum* or *P. nigrita* stimuli (Fig. 3, Table 1). The IC and Ls contribute to conspecific recognition and social behavior in other anurans and diverse taxa. In túngara frogs, the laminar subnucleus of the IC responds selectively to conspecific vocalizations (Hoke, 2004; Mangiamele and Burmeister, 2011). Likewise, the teleost homolog of the IC shows greater immediate early gene induction in response to conspecific vocalizations than to ambient noise in female midshipman fish (Mohr et al., 2018). In anurans, selectivity for conspecific calls may derive from time-decoding auditory neurons in the IC tuned to the temporal components of the conspecific call (Edwards et al., 2002; Rose et al., 2015; Gupta et al., 2021). An increase in the number of pS6-positive cells in this region may indicate the induction of a higher instantaneous spike rate in these neurons (Witharana et al., 2019). In the forebrain, the Ls broadly contributes to social behaviors. Anoles exposed to playback of an anole aggressive display show greater activity in the Ls than do control animals (Yang and Wilczynski, 2007). Similarly, guppies demonstrate increased neural activity in the teleost homolog of the Ls in both mating and aggressive social contexts compared with that in social isolation (Fischer et al., 2018). These results are consistent with our finding that sympatric male *P. feriarum* experience greater activation of the Ls in response to sympatric conspecific calls because anuran advertisement calls often convey both mating and aggressive information (Wells, 1977).

Evoked activity patterns in the Dp and Str point towards a role of spatial orientation and navigation in neural enhanced species recognition, likely indicating involvement in chorus structuring. In the toad *Rhinella arenarum*, the Dp shows increased activity during spatial navigation with visual cues (Sotelo et al., 2016). This region, along with the Str, provides key input to the Mp – the amphibian homolog of the mammalian hippocampus – in a proposed Mp-dependent spatial network (Sotelo et al., 2024). The amphibian Str is thought to be homologous to the mammalian caudate nucleus (Parent, 1986), which contributes to navigation in laboratory rodents (Whishaw et al., 1987) and humans (Iaria et al., 2003). In amphibians, the Str is a key structure for spatial orienting (Ruhl et al., 2016) and sound-directed movement (e.g. antiphonal calling and phonotaxis; Walkowiak et al., 1999). Neural activity in the Dp and Str specifically in response to the sympatric *P. feriarum* call suggests that reception of this stimulus induces behaviors involved in navigation and spatial orientation. Considering chorus frog breeding phenology, this finding is particularly relevant. To successfully breed, male *P. feriarum* must first locate and navigate towards a conspecific breeding chorus (which occur sporadically and explosively; Ethier et al., 2021) and then find a site in the chorus in which to call, while reducing spatial and acoustic interference with competing males and using the calls of other males as cues for timing their calls (Wilczynski and Brenowitz, 1988; Gerhardt et al., 1989; Brooke et al., 2000). Thus, evoked activation of cells in the Dp and Str likely contribute to navigational abilities of male *P. feriarum* at long (e.g. locating a chorus) and short (e.g. establishing a calling site within a chorus) distances.

### Functional connectivity among SDMN regions

Each sound-based stimulus resulted in a distinct pattern of coactivation among SDMN regions (Fig. 4, Table 2). Previous work has suggested that functional connectivity among brain regions, rather than the specialization of individual regions, better explain neural differences in response to distinct social contexts (Hoke et al., 2005; Teles et al., 2015). In the case of male *P. feriarum*, it seems that both specialization within and connectivity between SDMN nodes describe evoked neural activity differences. We found two significant pairwise correlations in neural activity among the brain regions we measured that were unique to the sympatric stimulus group: Dp–IC and Mp–IC (Fig. 4, Table S1). The activation and coactivation of pallial subregions in response to biologically relevant sound is consistent with work in túngara frogs that demonstrates auditory selectivity for conspecific vocalizations in several pallial subregions (Mangiamele and Burmeister, 2008). Because the IC shows both greater pS6 induction in response to the sympatric call and significant coactivation patterns unique to the sympatric stimulus, this region likely plays a key role in distinguishing conspecific versus heterospecific signals as well as familiar versus foreign conspecific signals. Together, the coactivation patterns we observed in response to the sympatric *P. feriarum* call suggest that coordinated neural activity between the auditory midbrain and the pallium contributes to neural enhanced species recognition.

We additionally found that the sympatric and heterospecific stimuli result in more similar coactivation patterns than any other stimulus pair, and that the heterospecific stimulus resulted in more significant pairwise correlations between regions overall. The sympatric and heterospecific stimuli coactivation matrices are more similar (*r*=0.186) than the other two pairwise comparisons (*r*=0.028 and *r*=0.057; Table 2), and the only shared significant regional correlations are shared between the sympatric and heterospecific stimulus groups (Fig. 4). In both stimulus groups, Acc–PAG, Dp–Mp and Mp–POA show significant coactivation (Fig. 4). The amphibian preoptic area acts as a key mediator integrating auditory (i.e. mating calls) and physiological cues (i.e. reproductive readiness; reviewed by Burmeister, 2017). Across diverse taxa, the PAG acts as a conserved neural substrate for courtship display (reviewed by Schwark et al., 2022) and, in amphibians, receives direct projections from the auditory midbrain (Luksch and Walkowiak, 1998). Evidence in the reptile Acc points towards a role in general social perception for this region in non-mammals (Yang and Wilczynski, 2007). The heterospecific call elicited many more unique significant pairwise correlations between regions than any other call type (Fig. 4). Especially considering our behavioral results, described below, this broad coactivation among SDMN nodes suggests that male *P. feriarum* uniquely perceive *P. nigrita* calls, perhaps because heterospecific calls may signal the timing and location of breeding events. Together, these results suggest neural triggers for sociosexual communication in response to both the sympatric and heterospecific calls.

### Similar behavioral responses to biologically distinct stimuli

We found significant neural differences, but little behavioral distinction, among male frogs exposed to differing acoustic stimuli. Frogs called less in the silence treatment than during any of the call stimuli (Fig. 5, Table 3), which validates our view that the frogs' behavioral responses were non-random. Most frogs, both while actively calling and while silent, spent a significant proportion of the trial time either in the central tub or perching on the edge of the tub orienting their heads to a speaker (Fig. 5, Table S2). Although this finding is superficially discordant with work in other species that finds that males will readily perform directed phonotaxis towards other male calls (e.g. Reichert and Gerhardt, 2014), our study suggests that increasing the sound intensity of a simulated call increases the likelihood of physical approach, whereas lower sound intensity levels are more likely to induce stationary antiphonal calling, as we observed here in male *P. feriarum*. We equilibrated our speakers to 75 dB to simulate a *P. feriarum* advertisement call at ∼1 m, which approximates a natural chorus environment with a calling neighbor. Therefore, the high proportion of time spent in and around the tub edge we observed may be due to males perceiving a non-intrusive calling neighbor, mirroring chorus spatial structuring. Future work will perform similar tests at higher and lower sound intensity levels to measure how this behavior changes at different perceived distances from calling neighbors.

Largely similar behavioral responses to distinct biological cues indicates that signal input and behavioral output are coded independently in the chorus frog brain (e.g. Fischer et al., 2018). Given that mixed species choruses can occur in sympatric *P. feriarum* populations, this apparently independent coding of social signal inputs and behavioral outputs may be adaptive. Male túngara frogs are more permissive towards heterospecific calls than females because males experience relatively relaxed selective pressure for species recognition owing to their lower reproductive investment (Bernal et al., 2007). Further, male túngara frogs and males of another tropical species (*Chiasmocleis shudikarensis*) respond behaviorally to sympatric heterospecific calls as they do to conspecific calls (Phelps et al., 2007; Fouquet et al., 2021). The latter species likely benefits from paying attention to heterospecific (*Trachycephalus coriaceus*) calls, because the presence of this species serves as a reliable signal for the proximity of a suitable breeding pond (Fouquet et al., 2021).

Our results show that male *P. feriarum* may distinguish between conspecific and heterospecific calls, as well as between allopatric and sympatric *P. feriarum* calls, at a neural level. If male *P. feriarum* can distinguish among the call types but still demonstrate similar behavioral responses, then our results point towards an adaptive basis for distinct social input that generates similar behavioral output. It may be beneficial for male *P. feriarum* to attend to any conspecific call (sympatric or allopatric), as well as the *P. nigrita* call, for two possible reasons. First, because *P. nigrita* are relatively more abundant than *P. feriarum* at the sympatric site we studied (Lemmon, 2009) and the two species' breeding seasons largely overlap (Ethier et al., 2021), heterospecific calls may serve as a conspicuous signal for the location and timing of an explosive breeding event for male *P. feriarum*. In this scenario, despite neural activity differences in response to conspecific and heterospecific calls, it benefits male *P. feriarum* to respond to calls in the same way, regardless of species identity, in order to locate and navigate to chorusing sites. A second, not mutually exclusive, alternative to this proposed explanation is that male *P. feriarum* respond in the same way to conspecific and heterospecific males because both are potential competitors. Interspecific hybridization does occur in sympatric areas (Anderson et al., 2023), so *P. feriarum* males may compete with the more abundant *P. nigrita* males not only for females but also for calling sites within a chorus. In both proposed explanations, the neural patterns we observed in male *P. feriarum* may result from shared neural circuitry for species recognition, which is under strong selection in females (Lemmon and Lemmon, 2010). In which case, sexually dimorphic neuromodulatory mechanisms may explain behavioral discrepancies between the sexes (see Mowrey and Portman, 2012 for review).

### Conclusions

Our findings represent the first step towards understanding the neural basis of conspecific species recognition in a system in which this trait contributes to ongoing diversification. This work lays the foundation for future studies that will examine sex differences and geographic variation (e.g. allopatric *P. feriarum*) in evoked neural activity in the focal species. We identified core brain regions and coactivation patterns among regions with navigational and sociosexual functions that are specifically responsive to the sympatric conspecific call in the sympatric *P. feriarum* brain. Although activity in these brain regions, as well as coactivity among key regions, represents a candidate neural basis for enhanced species recognition, we found that frogs behaviorally respond similarly to heterospecific and conspecific stimuli. This result suggests the independent coding of sexual signal input and behavioral output in the brain. Together, this work contributes to an understanding of how animals recognize conspecifics and the role of animal cognition in the speciation process.

## Supplementary Material

10.1242/jexbio.251686_sup1 Supplementary information
