# Supplementary material for: Neural and behavioral responses to reproductive signals in male chorus frogs
Source: J Exp Biol. 2026 Apr 20;229(8):jeb251686. doi: 10.1242/jeb.251686 (PMC13143210; doi:10.1242/jeb.251686)
Supplement: Supplementary information [file jexbio-229-251686-s1.pdf]

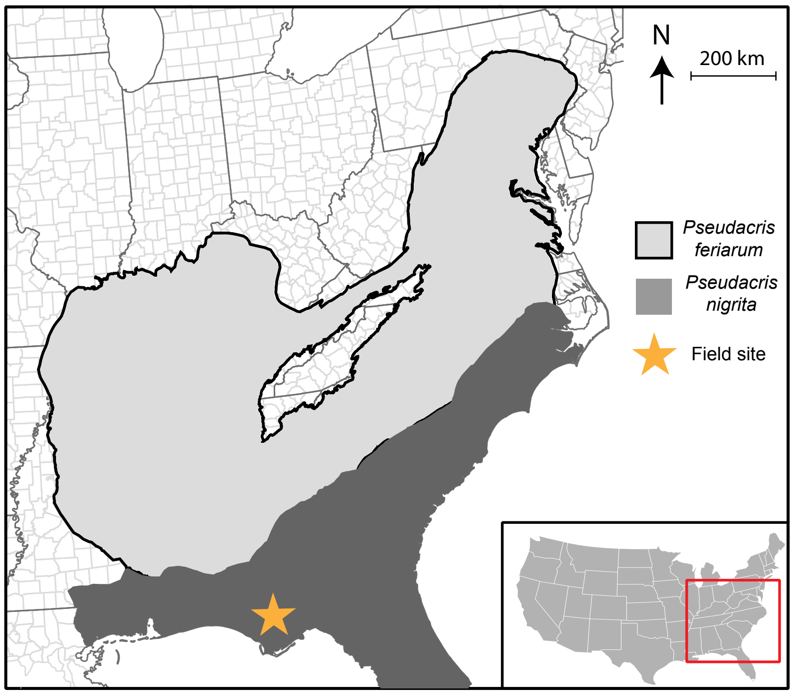

Fig. S1. Map of the southeastern United States with the ranges of *Pseudacris feriarum* and *P. nigrita* overlaid. The star indicates the field site where all animals were collected for this study (Liberty, Co., Florida). Inset map displays relative location.

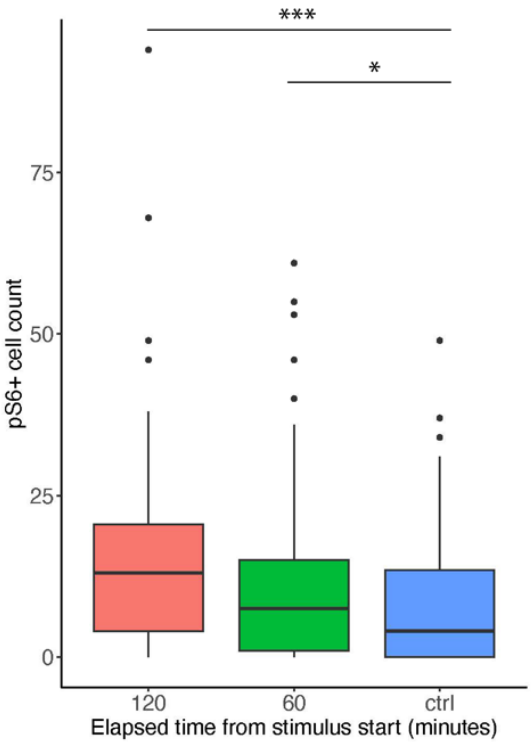

Fig. S2. The number of pS6-positive cells across all quantified brain regions varies by the elapsed time from the start of acoustic stimulation. The “ctrl” group received no acoustic stimulation (silence).

Journal of Experimental Biology • Supplementary information

Table S1. P-value matrices for correlations of ps6+ cell counts between 12 brain regions of frogs exposed to the (A) Sympatric *P. feriarum* stimulus, (B), Allopatric *P. feriarum* stimulus, and (C) heterospecific (*P. nigrita*) sound stimulus. Brain region abbreviations are the same as those in Table 1. These data are also displayed in Figure 4. Significant ($p<0.05$) p-values are underlined and indicate a correlation between the number of pS6-positive cells in a pair of brain regions.

(A)

	**Acc**	**POA**	**BST**	**Dp**	**PAG**	**IC**	**Ls**	**MeA**	**Mp**	**Str**	**PT**
**Acc**											
**POA**	0.871										
**BST**	0.515	0.963									
**Dp**	0.793	0.090	0.199								
**PAG**	0.017	0.507	0.722	0.596							
**IC**	0.519	0.146	0.198	0.008	0.869						
**Ls**	0.629	0.823	0.250	0.181	0.474	0.296					
**MeA**	0.897	0.439	0.599	0.498	0.573	0.615	0.627				
**Mp**	0.903	0.018	0.489	0.002	0.470	0.024	0.275	0.563			
**Str**	0.984	0.532	0.373	0.864	0.880	0.696	0.674	0.140	0.597		
**PT**	0.522	0.814	0.240	0.497	0.652	0.216	0.262	0.480	0.696	0.535	
**VH**	0.741	0.653	0.824	0.419	0.486	0.225	0.251	0.585	0.396	0.170	0.090

(B)

	**Acc**	**POA**	**BST**	**Dp**	**PAG**	**IC**	**Ls**	**MeA**	**Mp**	**Str**	**PT**
**Acc**											
**POA**	0.030										
**BST**	0.518	0.562									
**Dp**	0.891	0.364	0.310								
**PAG**	0.311	0.894	0.318	0.261							
**IC**	0.701	0.274	0.388	0.669	0.638						
**Ls**	0.472	0.196	0.524	0.130	0.314	0.954					
**MeA**	0.042	0.339	0.274	0.221	0.191	0.703	0.530				
**Mp**	0.405	0.526	0.927	0.705	0.763	0.470	0.018	0.762			
**Str**	0.104	0.627	0.271	0.110	0.474	0.807	0.862	0.069	0.320		
**PT**	0.472	0.339	0.367	0.648	0.191	0.533	0.530	0.352	0.479	0.894	
**VH**	0.336	0.051	0.206	0.410	0.508	0.076	0.111	0.944	0.392	0.768	0.622

(C)

	Acc	POA	BST	Dp	PAG	IC	Ls	MeA	Mp	Str	PT
**Acc**											
**POA**	0.303										
**BST**	0.451	0.138									
**Dp**	0.315	0.244	0.010								
**PAG**	0.018	0.035	0.138	0.050							
**IC**	0.959	0.856	0.709	0.990	0.582						
**Ls**	0.210	0.564	0.393	0.444	0.876	0.683					
**MeA**	0.255	0.630	0.869	0.690	0.512	0.428	0.111				
**Mp**	0.180	0.018	0.006	0.040	0.018	0.846	0.393	0.869			
**Str**	0.523	0.853	0.782	0.541	0.853	0.011	0.523	0.538	0.843		
**PT**	0.847	0.375	0.340	0.293	0.717	0.009	0.728	0.231	0.365	0.004	
**VH**	0.573	0.436	0.380	0.263	0.652	0.449	0.040	0.135	0.403	0.307	0.069

Journal of Experimental Biology • Supplementary information

Table S2. Descriptive statistics of scored behaviors. Abbreviations are the same as those in Table 1. All measurements are in seconds with standard deviation in parentheses. We cannot include data for the Silence group for positional behaviors that we scored relative to stimulus speaker, since this treatment did not have a stimulus-playing speaker.

Behavior	Sym mean	Allo mean	Het mean	Silence mean
**Calling duration**	135 (198)	101 (149)	87 (140)	2 (10)
**Duration in tub**	497 (142)	503 (128)	523 (124)	549 (142)
**Total time in motion**	24 (30)	25(29)	27 (52)	15 (23)
**Total time on stimulus side**	91 (124)	37(59)	30 (63)	NA
**Total time in stimulus pool**	54 (87)	43 (68)	10 (23)	NA
**Total time on stimulus edge**	22 (62)	2.6 (6.6)	17 (56)	NA
**Total time on opposite side**	26 (72)	34 (78)	54 (85)	NA
**Total time in opposite pool**	3.7 (12.3)	21 (64)	23 (56)	NA
**Total time on opposite edge**	22 (72)	13 (48)	29 (72)	NA

Table S3. AICc model selection for models including an effect of SVL, body mass, or trial number on behavior duration.

Model	K	Delta AICc	AICc weight	Log likelihood
**Null model**	32	0.00	1	-4060.13
**Trial number**	62	42.03	0	-4046.52
**SVL**	62	48.54	0	-4049.77
**Mass**	62	60.66	0	-4055.83

Journal of Experimental Biology • Supplementary information
